# An *In Vivo* Platform for Tumor Biomarker Assessment

**DOI:** 10.1371/journal.pone.0026722

**Published:** 2011-10-26

**Authors:** Elliot L. Servais, Kei Suzuki, Christos Colovos, Luis Rodriguez, Camelia Sima, Martin Fleisher, Valerie W. Rusch, Michel Sadelain, Prasad S. Adusumilli

**Affiliations:** 1 Division of Thoracic Surgery, Memorial Sloan-Kettering Cancer Center, New York, New York, United States of America; 2 Center for Cell Engineering, Memorial Sloan-Kettering Cancer Center, New York, New York, United States of America; 3 Department of Epidemiology and Biostatistics, Memorial Sloan-Kettering Cancer Center, New York, New York, United States of America; 4 Department of Clinical Laboratories, Memorial Sloan-Kettering Cancer Center, New York, New York, United States of America; 5 Department of Medicine, Memorial Sloan-Kettering Cancer Center, New York, New York, United States of America; Johns Hopkins Hospital, United States of America

## Abstract

Tumor biomarkers provide a quantitative tool for following tumor progression and response to therapy. However, investigations of clinically useful tumor biomarkers are time-consuming, costly, and limited by patient and tumor heterogeneity. In addition, assessment of biomarkers as indicators of therapy response is confounded by the concomitant use of multiple therapeutic interventions. Herein we report our use of a clinically relevant orthotopic animal model of malignant pleural mesothelioma for investigating tumor biomarkers. Utilizing multi-modality imaging with correlative histopathology, we demonstrate the utility and accuracy of the mouse model in investigating tumor biomarkers – serum soluble mesothelin-related peptide (SMRP) and osteopontin (OPN). This model revealed percentage change in SMRP level to be an accurate biomarker of tumor progression and therapeutic response – a finding consistent with recent clinical studies. This *in vivo* platform demonstrates the advantages of a validated mouse model for the timely and cost-effective acceleration of human biomarker translational research.

## Introduction

Serum or plasma tumor biomarkers provide reliable, easily measurable, and noninvasive indicators of cancer detection, disease progression, and therapy response. However, clinical investigations of biomarkers are constrained by patient heterogeneity and selection bias, particularly in the setting of rare malignancies such as malignant pleural mesothelioma (MPM) [1], [2]. MPM is an aggressive cancer of the pleural linings of the lungs and chest wall with an annual incidence of fewer than 3,000 cases in the US [3]. Clinical investigation of MPM biomarkers is hindered by disease rarity as well as a dismal median survival of 9–17 months even with the best available therapy [4], [5]. Despite over 450 publications investigating MPM biomarkers, no candidate MPM biomarker has become a standard of care.

Currently, the only biologic metric of response to MPM therapy is the use of radiological endpoints. Multiple serial radiographic measurements required to monitor therapy response are not only costly but also suffer from inter-observer variations due to the unique growth pattern of MPM as a pleural rind instead of discrete tumor nodule [6]. In addition, the standard Response Evaluation Criteria in Solid Tumors (RECIST) used to assess therapy response in solid tumors is inadequate for MPM because of the limitation of one-dimensional measurement for change in pleural tumor burden [7]. Although FDG-PET (fludeoxyglucose positron emission tomography) scans have been shown to correlate with therapy response [8], [9], this technique possesses shortcomings in interpretations including false-positive uptake in inflamed tissues. This highlights the need for biomarkers that can be utilized to provide a quantitative measure of disease progression and therapy response. Furthermore, the use of biomarkers offers a potential investigational tool to understand tumor biology and the efficacy of novel experimental therapies in pre-clinical animal models.

To investigate the utility of MPM biomarkers that are currently in clinical investigations, we utilized an orthotopic MPM mouse model. Our initial studies showed that commonly used MPM flank tumor models are unreliable for biomarker investigations due to unpredictable variations in serum levels as tumors grow larger and central necrosis occurs in late stages of disease. Likewise, in intraperitoneal models, accurate non-invasive quantification of tumor burden is limited due to the distribution of the tumor. In the current study, we developed a well-characterized orthotopic pleural animal model that closely resembles human MPM. We then utilized this model to investigate two candidate MPM biomarkers - soluble mesothelin-related peptide (SMRP) and osteopontin (OPN). In addition to allowing the investigation of candidate biomarkers for a rare malignancy, our study demonstrates the utility of mouse models to determine biomarker response to therapy.

## Materials and Methods

### Ethics Statement

All procedures were performed under approved Institutional Animal Care and Use Committee (IACUC) protocols (#08-01-001).

### Generation of mesothelin and GFP-Luciferase MPM cells

The human MPM cell line, MSTO-211H (American Type Culture Collection), was maintained RPMI-supplemented with 10% fetal bovine serum, 100 units/mL penicillin, and 100 ug/mL streptomycin in a 5% CO_2_ humidified incubator at 37°C. Green fluorescent protein (GFP)-Firefly luciferase fusion and human mesothelin (MSLN) genes were cloned into SFG retroviral vectors, which were then transfected into H29 packaging cell line using calcium phosphate mediated transfection protocol. MSTO-211H cells were plated in 24-well plates 24 hours prior to retroviral transduction. Media containing filtered virus was added to cells permeablized using 8 ug/mL polybrene (Sigma-Aldrich, MO). Cells were reinfected with freshly collected virus 24 hours later. The human MSLN variant 1 was isolated from the human MSLN-expressing ovarian cancer cell line OVCAR using TRIzol Reagent. RT-PCR synthesis of full length cDNA of human MSLN was performed using SuperScript™ III One-Step RT-PCR System with Platinum® *Taq* High Fidelity Kit. The 5′ primer is GAT CTA CAC AGA CCA TGG CCT TGC CAA CGG, including a Nco I site, and the 3′ primer is GCG CAG ATC TTA CGT ATC AGG CCA GGG TGG AGG CTA G, including a Bgl II site and a Sna BI site. Plasmid DNA was isolated, subcloned into the SFG retroviral vector, confirmed by sequencing, and used to stably transduce MSLN.

### Flow Cytometry

Flow activated cell sorting (FACS) was performed using FACSAria (BD Biosciences). Human mesothelin cell-surface expression was detected using a phycoerythrin-conjugated or allophycocyanin-conjugated anti-human mesothelin rat IgG_2A_ (R&D systems, MN). Flow cytometry for GFP and mesothelin expression was performed on LSRII cytometer (BD Biosciences) and analyzed using FlowJo (Tree Star) software.

### Generation of orthotopic MPM mouse model

Female SCID/beige and male athymic nude mice (6–10 weeks of age, Taconic Farms, NY) were used. Mice anesthetized with inhaled isoflurane and oxygen underwent direct intrapleural injection of tumor cells in 200 uL serum-free media via a right thoracic incision. Following inoculation, tumor take and progression occurred in >95% of mice. Mice were sacrificed when moribund as per IACUC guidelines. The details of our intrapleural xenografting technique have been previously described [10], [11], [12].

### Immunofluorescence

Immunofluorescence staining for angio- and lymphangiogenesis was performed using CD34 rat monoclonal (eBioscience) and LYVE-1 goat polyclonal (R&D Systems) antibodies, respectively. Immunofluorescencent detection was performed with Streptavidin-HRP D (Ventana Medical Systems), followed by incubation with Tyramide-Alexa Fluor 488 (Invitrogen) or Tyramide Alexa Fluor 568 (Invitrogen) for CD34 and LYVE-1, respectively. The protocols were established and experiments performed at the Molecular Cytology Core Facility, MSKCC, using Discovery XT automatic processor from Ventana Medical Systems.

### Quantitative bioluminescence imaging

*In vitro* bioluminescence imaging (BLI) standardization was performed using GFP-Firefly luciferase expressing MSTO-211H cells with and without MSLN expression. Cells were plated in 96-well tissue culture plates in serial dilution from 1.6×10^6^ to 2.5×10^4^ cells/100 µL/well. Twenty minutes after the addition of 100 µL D-Luciferin to each well (15 mg/mL; Caliper Lifesciences, MA), plates were imaged using the Xenogen IVIS 100 Imaging System (Caliper Lifesciences). BLI data were analyzed using Living Image 2.60 software. Cell number versus total BLI flux (photon/s) was evaluated by Pearson's correlation.

*In vivo* BLI in tumor-bearing mice was performed using a single intraperitoneal dose of 150 mg/kg D-Luciferin. Mice were imaged with the Xenogen IVIS 100 Imaging System 20 minutes following D-Luciferin injection and images acquired for 5–30 seconds depending on signal strength. BLI data were analyzed using Living Image 2.60 software; BLI signal from regions of interest (ROI) were reported as total flux (photons/s).

### Magnetic resonance imaging

Magnetic resonance imaging (MRI) was performed on mice anesthetized with 1% isoflurane in 20% Oxygen and imaged in a Bruker 4.7T USR scanner (Bruker Biospin Inc., Ettlingen, Germany) equipped with a 400 mT/m gradient coil and a 32 mm ID custom build birdcage resonator. Thoracic axial MRI images were acquired using a RARE fast spin-echo sequence with a repetition time (TR) of 1.7 s, echo time (TE) 40 ms and 12 averages. The acquisition was triggered by animal respiration to reduce respiration induced motion artifacts. The slice thickness was 0.7 mm and the in-plane image resolution was 117×156 mm. Tumor volumes (mm^3^) were measured by tracing tumor boundaries in each slice using Bruker ParaVision Xtip software (Bruker Biospin Inc., Ettlingen, Germany) and then calculated from the areas of tumor regions in each slice.

### Serum SMRP and OPN assay

Blood was collected in K_2_EDTA tubes, immediately centrifuged to separate serum, and stored at −20°C until assayed. Soluble mesothelin-related peptide (SMRP) was measured using an ELISA microplate sandwich assay (Mesomark® Fujirebio Diagnostics, Inc). The detection limit of this assay was 0.3 nM. Serum osteopontin (OPN) was measured using Quantikine® OPN immunoassay. The assay, performed per manufacturer's instructions, is a solid-phase ELISA employing a sandwich immunoassay to quantitate human OPN. Samples were measured in duplicate with standard controls.

### Chemo- and radiation therapy

Biomarker performance in evaluating therapy response was assessed in Nude Athymic male mice, in which orthotopic pleural tumors, expressing both GFP-Luciferase and human mesothelin, were established as described above. Tumors progressed for 22 days following inoculation at which time equivalent tumor burden was confirmed by BLI signal and mice were divided into experimental groups. Mice received either: (a) cisplatin (4 mg/kg IP weekly), (b) isolated thoracic radiation (20 Gy total in 5 fractions), (c) combination chemoradiation, or (d) no treatment. Isolated thoracic radiation was provided using the XRAD-320 (Precision X-ray, CT) 250 kVp, 12 mA, 0.25 mm Cu filtration in conjunction with lead barrier protection of the mouse peritoneal cavity. Treatment was provided for two weeks and the mice were monitored serially with BLI and serum SMRP assessment. Differences in BLI signal and SMRP levels between treatment groups were assessed by Kruskal-Wallis test using area under the curve analysis and Student's t-test, respectively. Predictive value of SMRP for survival following treatment was evaluated using analysis of maximum likelihood estimates and reported as hazard ratios.

## Results

### Quantitative bioimaging in a clinically relevant mouse model

**Figure 1:**
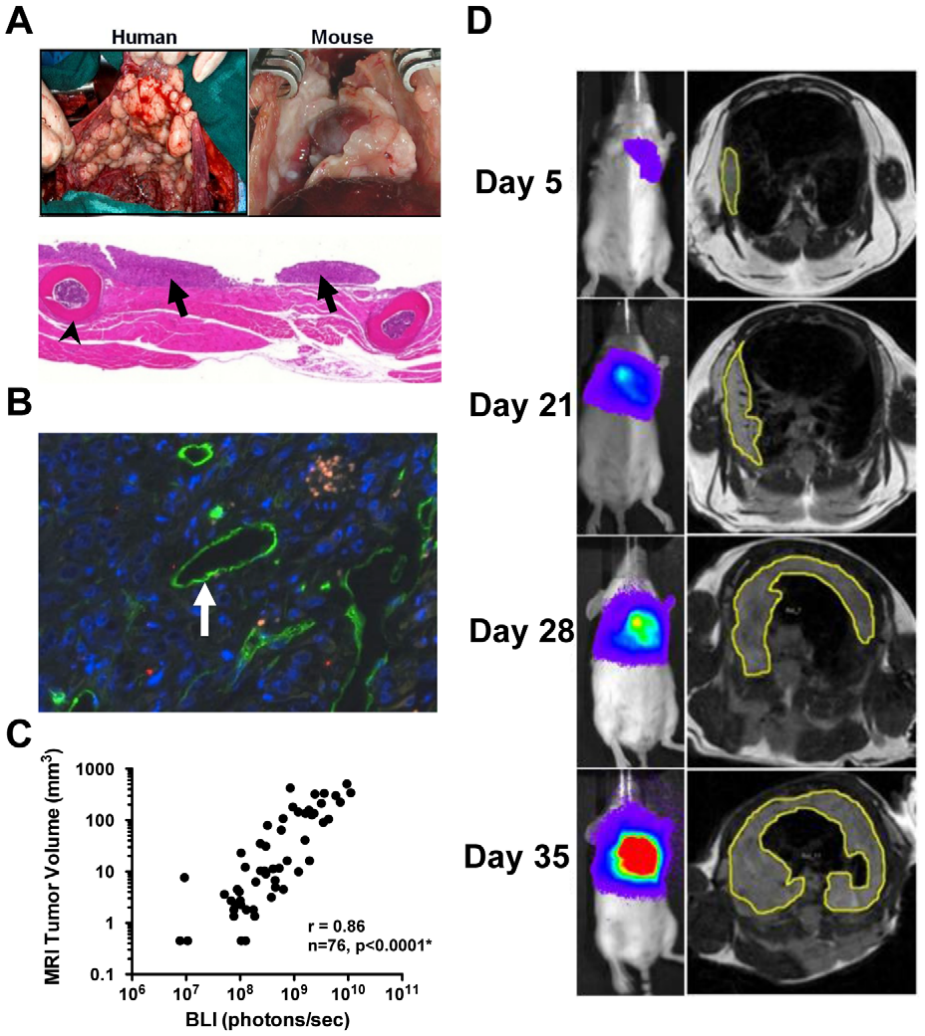
Orthotopic mouse model of MPM permits noninvasive, quantitative bioimaging of tumor progression. *A*, The mouse model of MPM recapitulates human disease and H&E staining of mouse chest wall (arrowhead at rib) with MPM demonstrates the pleural growth pattern of tumor (arrows) similar to human disease as an increased thickening of the pleural rind permitting quantitative BLI. *B*, Immunofluorescence staining demonstrates extensive angiogenesis (CD34 – green) in the advanced orthotopic pleural tumor permitting SMRP detection in serum. *C–D*, Serial BLI in pleural xenograft mice accurately reflects tumor burden as correlated with gold-standard assessment by serial volumetric MRI in the live mouse (r = 0.86, p<0.0001*). * Sample size corresponds to total number of measurements over time in a group of 14 mice; p-values are adjusted for within mouse clustering.

To evaluate biomarkers for MPM, we utilized an intrapleural mouse model. This model recapitulates the human tumor microenvironment grossly and histopathologically (Fig. 1A). In addition, the extensive vascularization of the MPM tumors in our model (Fig. 1B) facilitates detection of serum biomarker. This clinically relevant model provided an appropriate platform to investigate biomarkers for clinical translation. To non-invasively monitor tumor progression, we validated bioluminescent imaging (BLI) as an accurate, quantitative modality to assess tumor burden. We confirmed a linear correlation between number of mesothelioma cells (transduced to express firefly-luciferase) and BLI photon counts *in vitro* (Pearson r = 0.999, p<0.0001) and demonstrated that BLI can detect as few as 1×10^3^ tumor cells in the pleural space of mice (data not shown). We next demonstrated a strong correlation between bioluminescent flux and pleural tumor volume as determined by magnetic resonance imaging (MRI), the gold standard for tumor volume assessment [13] (r = 0.86, p<0.0001, adjusted for within mouse clustering; Fig. 1C and D). Accurate, quantitative BLI is possible in this model because tumor grows along the chest wall as a thickening of the pleural rind minimizing tumor depth and BLI signal attenuation (Fig. 1A). These experiments established that BLI in the orthotopic pleural model provides an accurate, non-invasive, serial, and quantitative evaluation of tumor burden.

### *In vivo* biomarker assessment of tumor progression

**Figure 2:**
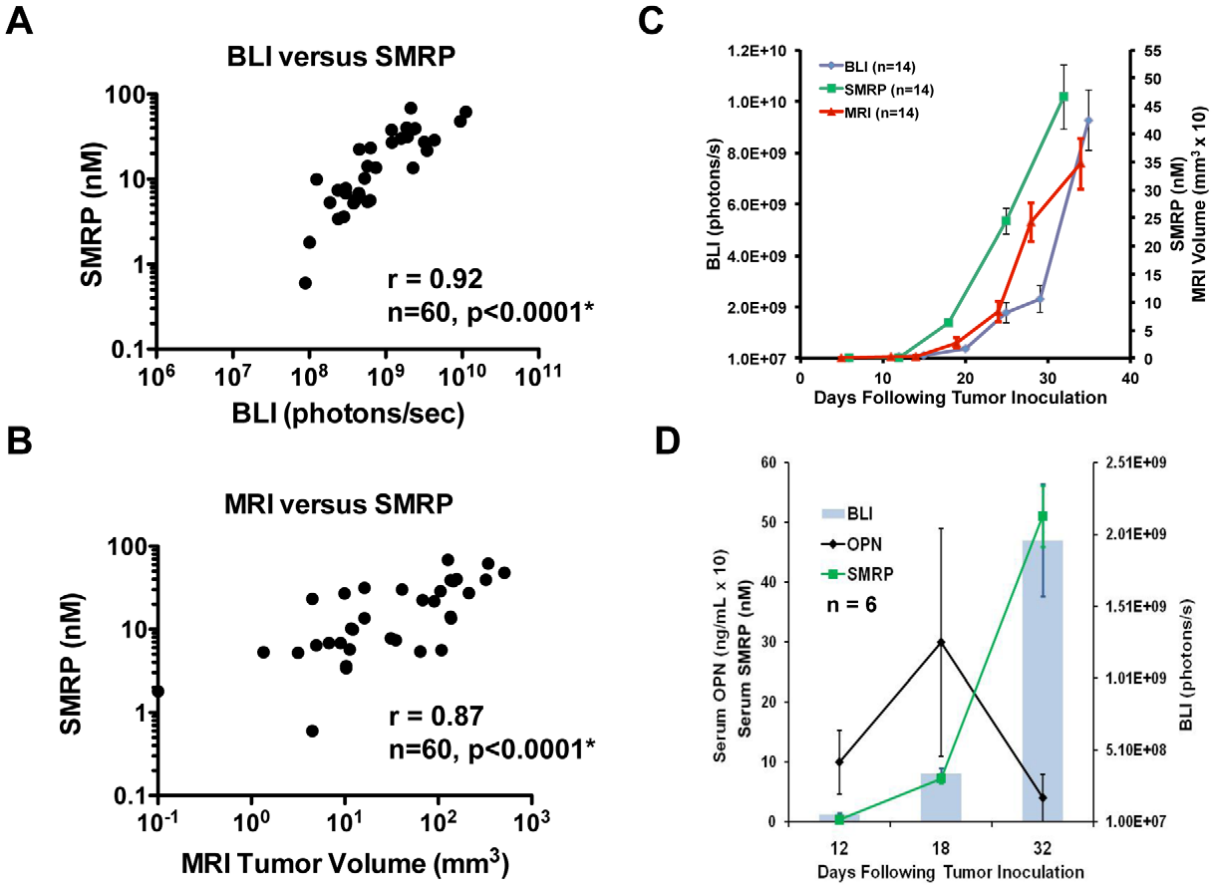
SMRP correlates with tumor volume and progression. *A–C*, SMRP increases with tumor progression and correlates with BLI signal (r = 0.92, p<0.0001) and tumor volume by MRI (r = 0.87, p<0.0001). *D*, The mouse model demonstrates that SMRP, but not OPN, correlates with tumor progression in MPM with less variability, as also observed in patients.

We next sought to evaluate the candidate mesothelioma tumor biomarker, SMRP. SMRP is a cleavage product of the cell-surface protein mesothelin, overexpressed by epithelioid MPM (the most common MPM subtype), and has shown promise as a tumor biomarker [3], [14], [15], [16]. We first confirmed sustained mesothelin expression in the orthotopic MPM model by immunohistochemistry and flow cytometry even at advanced stages of disease (data not shown). We observed that SMRP in the pleural MPM model correlated with tumor burden and progression as confirmed by MRI tumor volume and BLI signal (Fig. 2A–C). We simultaneously evaluated serum OPN, another candidate serum MPM biomarker. Using the aforementioned quantitative imaging parameters, serum OPN levels neither correlated with tumor burden (Fig. 2D) nor SMRP level. Based on these observations, we only evaluated SMRP in subsequent experiments.

### *In vivo* biomarker assessment of therapy response

**Figure 3:**
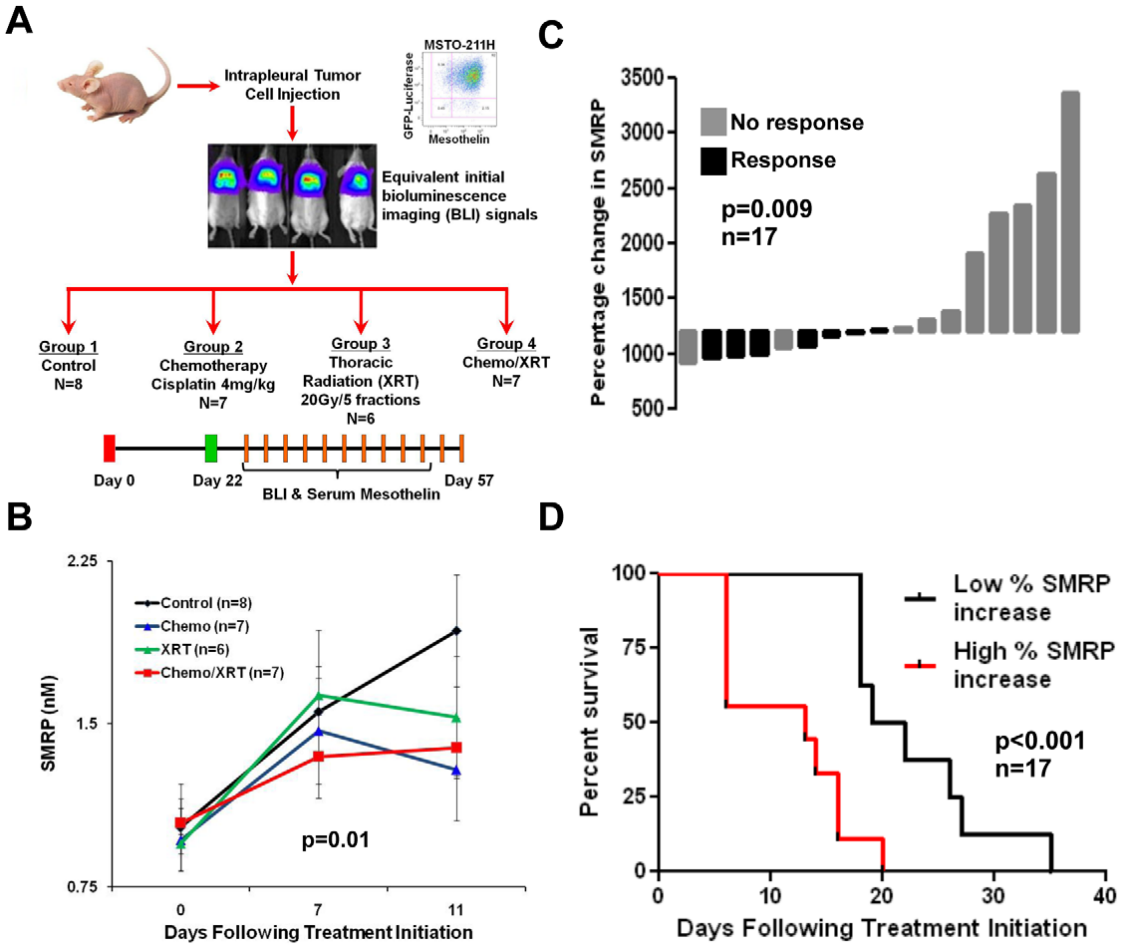
SMRP is a biomarker of therapy response. *A*, Nude athymic mice xenografted with human MPM tumors were treated with either: cisplatin (4 mg/kg IP weekly), isolated thoracic radiation (20Gy total in 5 fractions), combination chemoradiation, or no treatment. Differential response to therapy was monitored by BLI signal and serum SMRP. *B*, Serum SMRP was significantly decreased in treatment groups compared to controls as reflected by a lower increase in SMRP over the treatment period (Wilcoxon test, p = 0.01). *C*, Treatment response following therapy, defined as less than a 2-fold increase in BLI signal over the treatment period, was associated with lower percent increase in SMRP as shown by the waterfall plot (p = 0.009). Note that all treatment groups had increasing tumor despite therapy, reflecting the limited effectiveness of standard therapies against MPM. Therefore, “response” versus “no response” is relative to the median percentage increase in SMRP (a 12-fold increase) for the entire cohort. *D*, Additionally, mice with low percent SMRP increase (less than the group median) demonstrated improved survival following therapy (p<0.001).

To examine the importance of a serum biomarker in predicting therapy response in MPM, we evaluated serum SMRP levels following multimodality treatment of mice with orthotopic pleural tumors. Immunodeficient nude athymic mice were engrafted with human MPM tumor cells intrapleurally and then treated for two weeks with multimodality therapy (Fig. 3A). Mice were monitored using serial serum SMRP, BLI, and followed for survival. We noted a significant decrease in serum SMRP level in the treatment groups compared to controls. Although SMRP increased during chemoradiation treatment cycles (days 0–11), the rate of increase was significantly decreased (slope of 0.04 versus 0.21 for controls, Wilcoxon test, p = 0.01; Fig. 3B). Decreased serum SMRP correlated with slowed tumor progression as reflected in BLI signal following treatment compared to control mice (data not shown). In addition, elevated serum SMRP was associated with an increased risk of death (hazard ratio = 4.5, 95% CI 1.53–13.1). Using this mouse model we demonstrated that the percentage change in SMRP levels correlates with survival and response to therapy (defined as <2-fold increase in BLI signal over the treatment period). Following treatment, the percent increase in SMRP level was significantly lower in mice that responded to therapy compared to non-responders (10-fold *vs*. 17-fold increase, p = 0.009, Fig. 3C). Mice with a lower percent increase in SMRP (less than the group median) demonstrated improved survival compared to mice with a higher percent SMRP increase (p<0.001, Fig. 3D).

### Biomarker evaluation of novel therapies

**Figure 4:**
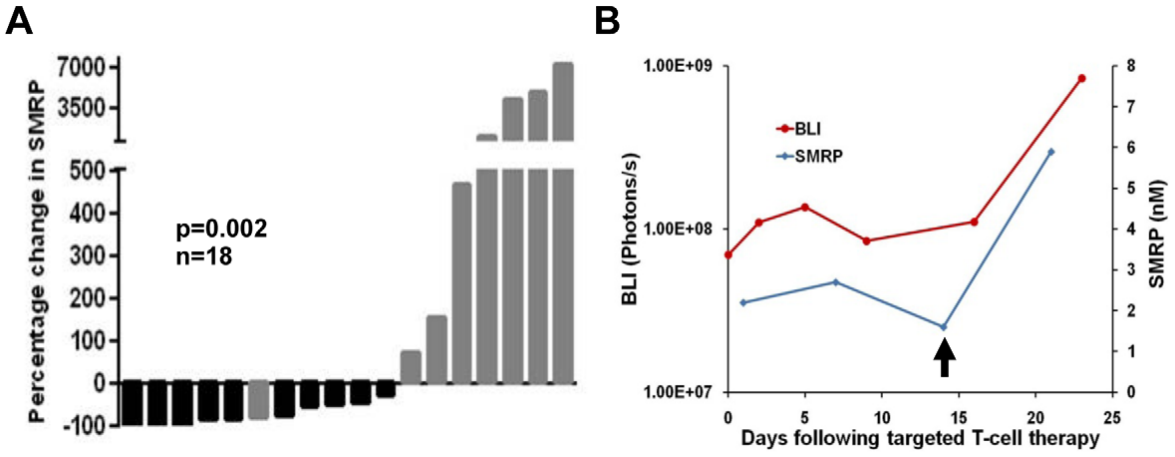
Utility of mouse model in evaluating biomarker response to therapy. *A*, Treatment response following T-cell immunotherapy, MPM-targeted versus control, defined as less than a 2-fold increase in BLI signal over the treatment period, was associated with percentage change in SMRP as illustrated by the waterfall plot (0.62-fold decrease vs. 11-fold increase, p = 0.002). *B*, SMRP sensitively detected early tumor relapse (arrow) following a single low-dose targeted therapy as indicated by a concomitant rise in BLI signal and serum SMRP.

Having evaluated the use of SMRP in the mouse model to detect therapeutic response following standard therapies, we next assessed the utility of the model in evaluating the efficacy of an emerging targeted therapy. Our laboratory is developing a targeted T-cell immunotherapy for MPM based upon our experience with genetically modified targeted human T cells [17]. Treatment response following MPM-targeted T-cell therapy versus control (unpublished observations), as defined as <2-fold increase in BLI signal over the treatment period, was associated with percentage change in SMRP (0.62-fold decrease vs.11-fold increase, p = 0.002, Fig. 4A). This noninvasive biomarker assessment has guided the dosing schedule in our ongoing immunotherapy experiments. Serum SMRP was a sensitive indicator of tumor progression following a response to an initial treatment with targeted T cells (Fig. 4B). These experiments demonstrate that serum SMRP is a sensitive, noninvasive marker of tumor response to therapy and relapse.

## Discussion

In summary, we have described an *in vivo* platform to investigate serum tumor biomarkers by combining quantitative bioimaging with an orthotopic mouse model. This report demonstrates that an orthotopic MPM mouse model provides a convenient and powerful platform to systematically evaluate biomarkers for monitoring tumor burden, disease progression, and therapeutic response – a process that is hindered in heterogeneous patient populations, particularly with rare diseases such as MPM. We have shown that this mouse model allows for accurate quantitative BLI and MRI and that serum SMRP, but not OPN, provides a sensitive biomarker of disease in MPM. The results of this study parallel the recently published clinical observations for SMRP and OPN in patients [16], [18], which demonstrate superiority of serum SMRP and limited utility of serum OPN as biomarker in MPM. It is notable that these preliminary clinical data [18] were obtained from a sample of 41 patients, taking 21 months to accrue, in contrast to our pre-clinical study requiring less than six months. In this mouse model, SMRP provides an early indication of therapeutic response during ongoing chemotherapy and radiation treatment. These results demonstrate the utility of biomarker assessment to detect therapeutic response at a cellular level, in contrast to standard imaging modalities, which detect macroscopic responses occurring at a later stage. This is particularly important for MPM, in which assessment of disease progression or therapy response is currently performed by serial CT [6] or PET scans [8], [9].

With the rise of ‘personalized medicine’, the value of biomarkers has become even more important in monitoring the efficacy of targeted therapies. We have shown that investigational therapies can be evaluated by correlating response to biomarkers in the mouse model providing a quantifiable parameter of response in addition to survival and helping to guide novel therapy treatment schedules. In developing such preclinical models, our study highlights the importance of utilizing orthotopic tumor models that recapitulate the tumor microenvironment and the use of validated correlative modalities to assess tumor progression, such as quantitative bioimaging. Furthermore, this study demonstrates the utility of orthotopic mouse models in preclinical studies of tumor biomarkers, biology, and therapy evaluation. In conclusion, we have developed an *in vivo* platform, which will allow for preclinical investigations of candidate biomarkers and can accelerate the design of biomarker clinical trials and ultimately translation to clinical application.
